# Harvest Pressure on Coastal Atlantic Cod (*Gadus morhua*) from Recreational Fishing Relative to Commercial Fishing Assessed from Tag-Recovery Data

**DOI:** 10.1371/journal.pone.0149595

**Published:** 2016-03-09

**Authors:** Alf Ring Kleiven, Albert Fernandez-Chacon, Jan-Harald Nordahl, Even Moland, Sigurd Heiberg Espeland, Halvor Knutsen, Esben Moland Olsen

**Affiliations:** 1 Institute of Marine Research, Flødevigen Marine Research Station, Nye Flødevigveien 20, N-4817 His, Norway; 2 Institute of Marine Research, Matre Research Station, N-5984 Matredal, Norway; 3 Centre for Ecological and Evolutionary Synthesis (CEES), Department of Biology, University of Oslo, PO Box 1066 Blindern, N-0316 Oslo, Norway; 4 Centre for Coastal Research, Department of Natural Sciences, Faculty of Science and Engineering, University of Agder, N-4604 Kristiansand, Norway; Institut Maurice-Lamontagne, CANADA

## Abstract

Marine recreational fishing is a popular outdoor activity. However, knowledge about the magnitude of recreational catches relative to commercial catches in coastal fisheries is generally sparse. Coastal Atlantic cod (*Gadus morhua*) is a target species for recreational fishers in the North Atlantic. In Norway, recreational fishers are allowed to use a variety of traps and nets as well as long-line and rod and line when fishing for cod. From 2005 to 2013, 9729 cod (mean size: 40 cm, range: 15–93 cm) were tagged and released in coastal Skagerrak, southeast Norway. Both high-reward (NOK 500) and low-reward tags (NOK 50) were used in this study. Because some harvested fish (even those posting high-reward tags) may go unreported by fishers, reporting rates were estimated from mark-recovery models that incorporate detection parameters in their structure, in addition to survival and mortality estimates. During 2005 to 2013, a total of 1707 tagged cod were recovered and reported by fishers. We estimate the overall annual survival to be 33% (SE 1.5). Recreational rod and line fishing were responsible for 33.7% (SE 2.4) of total mortality, followed by commercial fisheries (15.1% SE 0.8) and recreational fixed gear (6.8% SE 0.4). Natural mortality was 44.4% (SE 2.5) of total mortality. Our findings suggest that recreational fishing—rod and line fishing in particular—is responsible for a substantial part of fishing mortality exerted on coastal cod in southern Norway.

## Introduction

Recreational fishing, herein defined as all fishing activities not conducted for commercial and subsistence purposes (see [1] for further definitions), is a popular activity globally [2]. For some coastal species of fish and crustaceans, recent studies show that recreational fishing may be responsible for a substantial part of the total fishing mortality [2,3,4,5]. For the majority of harvested species, little is known about the relative importance of recreational fishing versus commercial fishing. However, if recreational fishing is significant, ignoring these catches may lead to mismanagement of fish stocks [6,7].

Atlantic cod (*Gadus morhua*) is a popular target species both for recreational and commercial fishers in Norway. In a recent study, Vølstad et al. [8] showed that this species made up the majority of fish caught (in weight) in the Norwegian marine tourist fishing industry nationwide. Herein, we use unique information from a tagging study on Atlantic cod conducted along ∼100 km of Skagerrak coastline from 2005–2013. Reporting letters supplied by fishers who have recovered tagged cod were used to analyse the catch ratio between commercial and recreational fishers as well as the fishing mortality exerted by the two groups. Our findings suggest that the recreational fishery, in which rod and line dominate, may be responsible for the greater part of fishing mortality exerted on coastal cod in this region.

## Materials and Methods

### Study species

**Fig 1:**
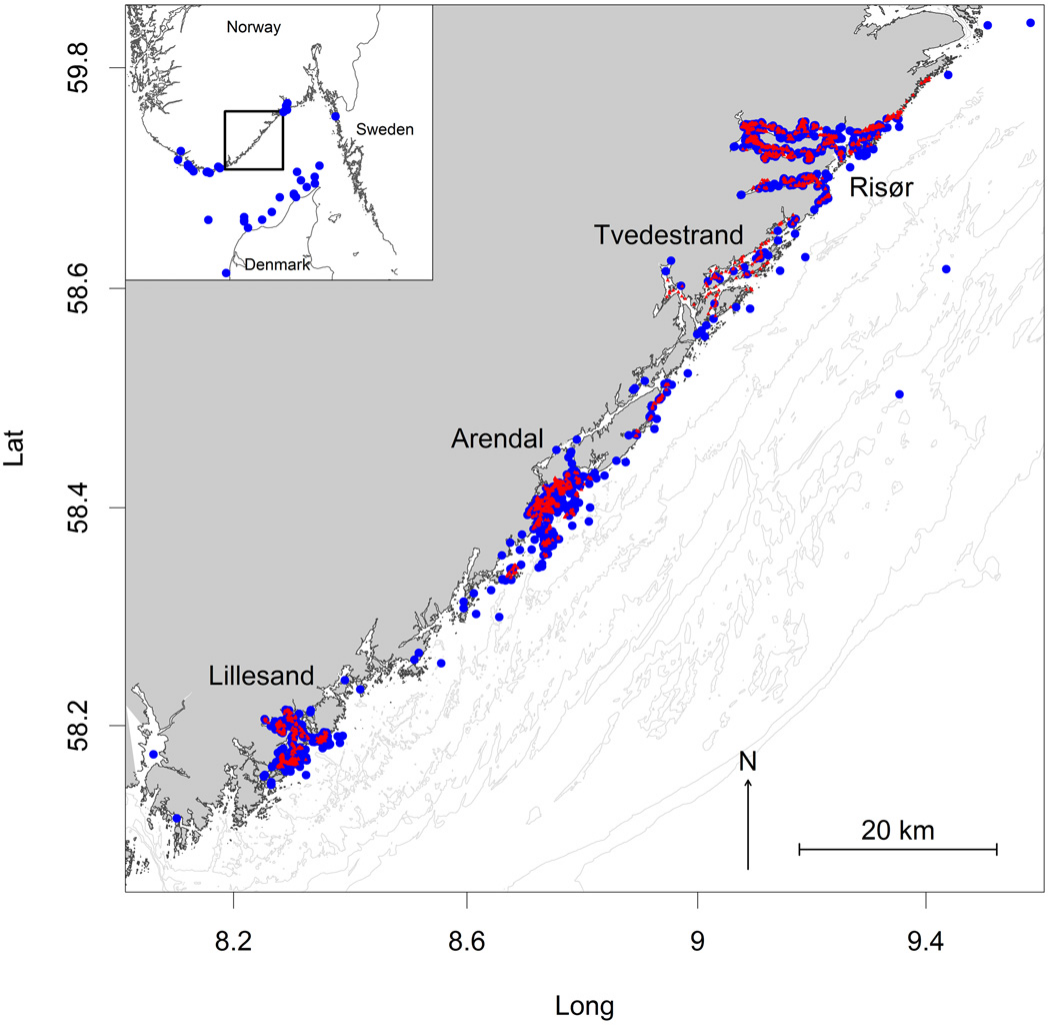
Map of study area (Aust-Agder County, Skagerrak) including cities and sampling area. Red dots: sites where cod was tagged. Blue dots: recapture sites.

The Atlantic cod is a large-bodied top predator and key species in coastal North Atlantic ecosystems [9,10]. It is also an important table fish, and many of the formerly large populations have been severely reduced by overfishing [10,11]. In Skagerrak (our study area; see Fig 1) there is evidence for local populations of cod separated by as little as 30 km of coastline [12,13,14], associated with inshore spawning behaviour and limited dispersal of eggs, juveniles and adults [15,16,17, 18]. Skagerrak coastal cod typically grow 10–15 cm per year and matures at an age of 2–3 years at a body length of 30–50 cm [19,20,21]. There is considerable fishing pressure on local populations [22,23]. Recreational fishers may fish with traps, pots, gill nets and long line, as well as rod and line. There are no licence requirements and cod above 40 cm (minimum legal size) can be fished all year round. Commercial fishers in Skagerrak catch cod as a direct target species as well as by-catch in a set of other fisheries (e.g., prawn trawling and wrasse fishery). Overall, the population density of cod in Skagerrak has remained at a low level since the 1980s [24,25].

### Study system

This study was conducted on the Norwegian Skagerrak coast, spanning the ∼100 km coastline of Aust-Agder County (Fig 1). This convoluted coastline contains several smaller fjords, archipelagos and a narrow shelf sloping down to the 500–700 meter deep Norwegian trench. The total coastal sea area shallower than 100 m amounts to 586 km^2^. Sea surface temperature may reach 20°C during summer (July-August) and drop below 0°C during winter (January-March). The study area harbours five small cities, with populations ranging between 2,300 and 43,000. In addition, a substantial proportion of the residents live scattered along the coast and on islands. The area is a popular tourist destination with a high concentration of summer houses.

### Data collection

Cod were captured by the authors in fyke nets (traps) and tagged during April − July, 2005 − 2013. A key assumption in mark-recapture-recovery studies is that tagged fish adequately represents the study population [26]. Following the recommendation by Pollock et al. [27], our study was designed so as to tag a smaller number of fish at multiple sampling locations within the study area, rather than tagging a larger number of fish in a few sites. This was achieved by (1) placing traps in all five municipalities along the Aust-Agder coast, and (2) by distributing traps across a range of habitats from the innermost sheltered fjord areas to the outermost exposed rocky habitats around islands bordering the open ocean. Fyke nets were set in shallow water (1 − 10 m) and soak time was usually 1–7 days (range 1 − 25 days). Fishing effort ranged from 490 − 2156 trap hauls among years (Table 1).

**Table 1:** Summary of raw data by year. Effort, number of trap deployments; Days, number of days in which traps were deployed; N_H_, number of high reward tags/ cod released; N_L_, number of cod released with low reward tags; L, range in body length (cm) of released cod, N_Recov_, number of high and low reward tags/ cod recovered.

Year	Effort	Days	N_H_	N_L_	L	N_Recov_
2005	*	*	153	1387	25–93	198
2006	1648	9615	168	1601	25–83	365
2007	1909	10299	355	1437	20–88	429
2008	1683	11057	322	1328	16–81	377
2009	860	7296	84	354	22–84	152
2010	913	3955	128	563	20–87	111
2011	493	855	110	86	21–69	122
2012	490	2863	313	794	25–80	143
2013	495	2482	122	429	25–73	56

*Not registered

Total length of all captured cod was measured to the nearest cm. Individuals, mostly > 250 mm, were tagged in the musculature at the base of the dorsal fin with traditional T-bar tags (Hallprint) with printed information containing a unique tag number, return address and reward. We used the high-reward tagging method [27,28] to estimate tag reporting rate. Specifically, every fifth fish (every tenth fish during 2005 − 2006) received a pink 500 NOK (≈ 80 €) high-reward tag, while the remaining fish received yellow 50 NOK (≈ 8 €) low-reward tags. The cod was released at the site of capture immediately after tagging, and the whole procedure from fyke net haul to release would usually take less than five minutes.

Commercial landings of cod in the area were 76 tons in 2012 (non-trawlers under 12 meters). Since the study focused on coastal Atlantic cod, prawn trawlers and boats larger than 12 meter were not included in the landing statistics.

### Recapture returns

Upon returning tags, most fishers reported the date, position of capture, type of fishing gear used as well as their home postal address. Based on this information, we defined three groups of recreational fishers: (1) local residents were recreational fishers living in the county of Aust-Agder and the neighbouring municipalities in other counties (Kristiansand in Vest-Agder county and Kragerø in Telemark county), (2) Norwegian tourists were recreational fishers living in Norway exclusive from local residents, and (3) foreign recreational fishers were fishers reporting an address outside Norway. Commercial fishers were identified based on personal knowledge as well as the Norwegian national fisher registry. Further, we divided catches by three groups of gear in the recreational fishery; i) rod and line ii) fixed gear (such as pots, traps and gill nets) and iii) unspecified gear (when the recapture reporting letter did not include type of gear used).

### Data analysis

The field records of tagged individuals were used to build up a 9-year mark-recovery data set that contained, for each sampling occasion, information on whether the individual was tagged or recovered and the cause of death (if recovered). This information was formatted and analyzed under a multi-event modelling approach [29]), a recently developed analytical framework that links our field observations to a series of underlying individual states defined in the model structure (see below and S1 Text).

**Fig 2:**
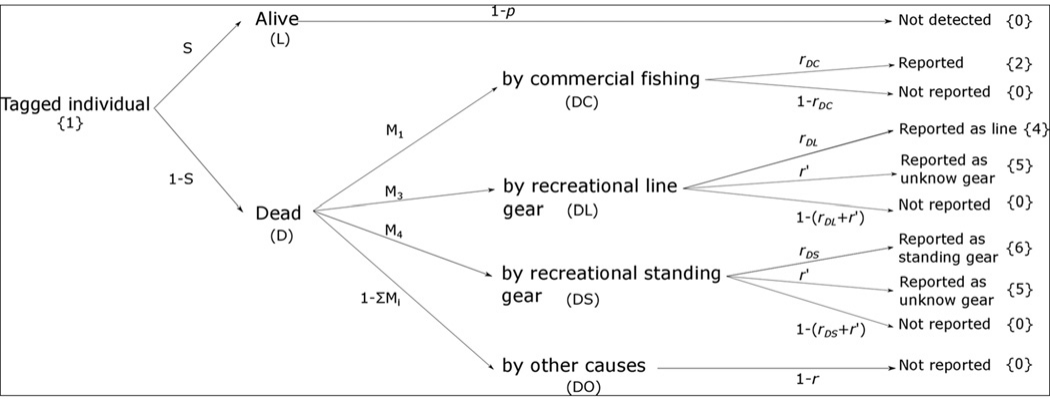
Diagram showing the model pattern used in the analysis of the mark-recovery data. Each step represents a different model parameter or transition probability between states and the whole sequence links both ecological (*S*,*M*) and observational processes (*p*,*r*) to the different events contained in the individual encounter histories (the numbers between brackets).

Our field data consist of multiple observations or “events” that were codified in the dataset as follows: not encountered (0), captured and marked for the first time (1), reported dead by commercial fisher (2), reported dead by recreational fisher using hook and line (3), reported dead by recreational fisher using fixed gear (4) and reported dead by recreational fisher using unknown gear (5). From this set of events, we estimated the proportion of fish mortality from different causes by constructing a model pattern based on transition matrices that linked the observed events to transitions between possible underlying states in which individuals may be found at a given sampling occasion (Fig 2). We considered that individuals can move among 5 states: alive (“L”), dead by commercial fisheries (“DC”), dead by recreational hook and line (“DL”), dead by recreational fixed gear (“DS”) and dead by other causes (“DO”). An extra unobservable dead state (†) was also included in the model definition to distinguish the observed recoveries or “newly dead” individuals from the unobservable “long-time dead” ones (see [30]). This classification allows the proportion of deaths associated with the different mortality causes to be estimated, and also the calculation of tag reporting rates for recovered fish (see below). Between each sampling occasion, fish can change state according to the transitions shown in Fig 2. The probabilities associated with each change of state are defined in the full transition matrix (**Φ**), which can be written as:

$$\Phi =\Phi =\left(\begin{array}{ccccccc} & L & DC & DL & DS & DO & †\\ L & S & M_{1}(1-S) & M_{2}(1-S) & M_{3}(1-S) & \left(1-M_{1}-M_{2}-M_{3})(1-S\right) & 0\\ DC & 0 & 0 & 0 & 0 & 0 & 1\\ DL & 0 & 0 & 0 & 0 & 0 & 1\\ DS & 0 & 0 & 0 & 0 & 0 & 1\\ DO & 0 & 0 & 0 & 0 & 0 & 1\\ † & 0 & 0 & 0 & 0 & 0 & 1 \end{array}\right)$$

where

*S*: the annual survival probability.

*M*_*1*_: the probability of death due to commercial fisheries given that an animal has died.

*M*_*2*_: the probability of death due to recreational hook and line given that an animal has died.

*M*_*3*_: the probability of death due to recreational fixed gear given that an animal has died.

These model parameters could be estimated separately by splitting the full transition matrix into a 2-step series of transition matrices representing survival and cause-specific mortality processes respectively (S1 Text). Our model pattern assumes that ecological processes occur before the observational ones, with survival being the first step in our sequence of transition matrices. If an individual die, it can transit to several dead states (Fig 2), thus allowing the estimation of the proportions of deaths associated with different mortality factors (*M*_*1*_, *M*_*2*_, *M*_*3*_). Finally, the third and last step corresponds to the observational process and allows us to estimate event probabilities. Matrix **E** shows the event probabilities that link the biological states (rows) with the observations (columns).

$$E=E=\left(\begin{array}{ccccccc} & 0 & 1 & 2 & 3 & 4 & 5\\ L & 1-p & p & 0 & 0 & 0 & 0\\ DC & 1-r_{DC} & 0 & r_{DC} & 0 & 0 & 0\\ DL & 1-r_{DL}-r' & 0 & 0 & r_{DL} & 0 & r'\\ DS & 1-r_{DS}-r' & 0 & 0 & 0 & r_{DS} & r'\\ DO & 1 & 0 & 0 & 0 & 0 & 0\\ † & 1 & 0 & 0 & 0 & 0 & 0 \end{array}\right)$$

where

*p*: the recapture probability of a marked animal that is alive.

*r*_*DC*_: the reporting probability of a marked animal dead by commercial fisher.

*r*_*DL*_: the reporting probability of a marked animal dead by recreational fisher using hook and line.

*r*_*DS*_: the reporting probability of a marked animal dead by recreational fisher using fixed gear.

*r’*: the reporting probability of a marked animal dead by recreational fisher using unknown gear.

Events 1 to 4 are directly linked to model states “L”,”DC”,”DL” and “DS” (i.e., they can only happen in these states) but event “0” (not encountered) arises from imperfect detection (see also Fig 2) and can be related to any possible underlying state in our probabilistic model. Event “5” (reported dead by recreational fisher using unknown gear) is linked to both “DL” and “DS” states and shows an associated probability different from the other event probabilities (*r’*; see above and Fig 2); this allows incorporating uncertainty into the model and to more robustly estimate the mortality proportions associated with recreational hook and line and fixed gear types. Given that only deaths caused by fishers can be reported, the state “DO” is not observable and thus it can only be linked to event “0” (see also Fig 2).

Multi-event models were built and fitted to the data using the program E-SURGE [31], but prior to the model selection process, a Goodness-of-fit (GOF) test was conducted to check if our data met the assumptions of a departure model that considers all parameters to be state and time dependent, namely the Arnason-Schwarz (AS) model [32]. GOF tests were performed using U-CARE [33]), a statistical programme designed to check whether the assumptions of this departure model are met. However, in our case, we followed a more conservative approach and we fitted a reduced version of the “AS” model that considered only 2 states (“alive” and “dead by fisher”), as unobservable states cannot be handled by U-CARE (for a similar approach see [33]). In order to scale model deviances and correct for remaining sources of lack of fit, an overdispersion coefficient or ĉ (calculated as the sum of chi-square results for each test divided by the total number of degrees of freedom) was introduced when performing the analysis in E-SURGE.

Model selection was based on the Akaike’s information criterion corrected for overdispersion (QAIC) and we retained as good candidate models those showing the lowest QAIC values [34]. The model selection process departed from a general model considering time effects in survival (*S*) and mortality parameters (*M*_*1*_, *M*_*2*_, *M*_*3*_) and reward effects on each reporting probability (*r*_*DC*_, *r*_*DL*_, *r*_*DS*_ and *r’*). We treated reward as a group effect, with two levels: high (500 NOK) and low (50 NOK). This statistical framework allowed us to obtain maximum likelihood estimates of reporting rates for each tagging group without the need to assume 100% reporting rates for high reward tags. The model selection process departed from a general model considering time effects in survival and mortality parameters and reward effects on each reporting probability. In addition, because we already knew that the quality of the reporting had improved towards the end of the study, we considered two periods in the probability of reporting deaths due to unknown gear: an early period (2006–2009) and a late period (2010–2013). Model selection consisted of progressively simplifying this structure by removing time effects on “S” and “M” parameters, until the best structure (the one with the lowest QAIC) was found.

### Ethics statement

The fishery for Atlantic cod in Norway is a managed fishery, conducted by both commercial and recreational fishers. The handling procedures of Atlantic cod were in accordance with treatment of animals through the Norwegian law of animal welfare (Dyrevelferdsloven 112 http://www.lovdata.no. Accessed 2013 Oct 29th). At the beginning of the study (2005–2006) there was no need for additional clearance to conduct the tagging procedures. However, from 2007 the handling and tagging of Atlantic cod were permitted by the Norwegian Animal Research Authority (Forsøksdyrutvalget, reference 2006/13686 and 07/52921).

## Results

**Fig 3:**
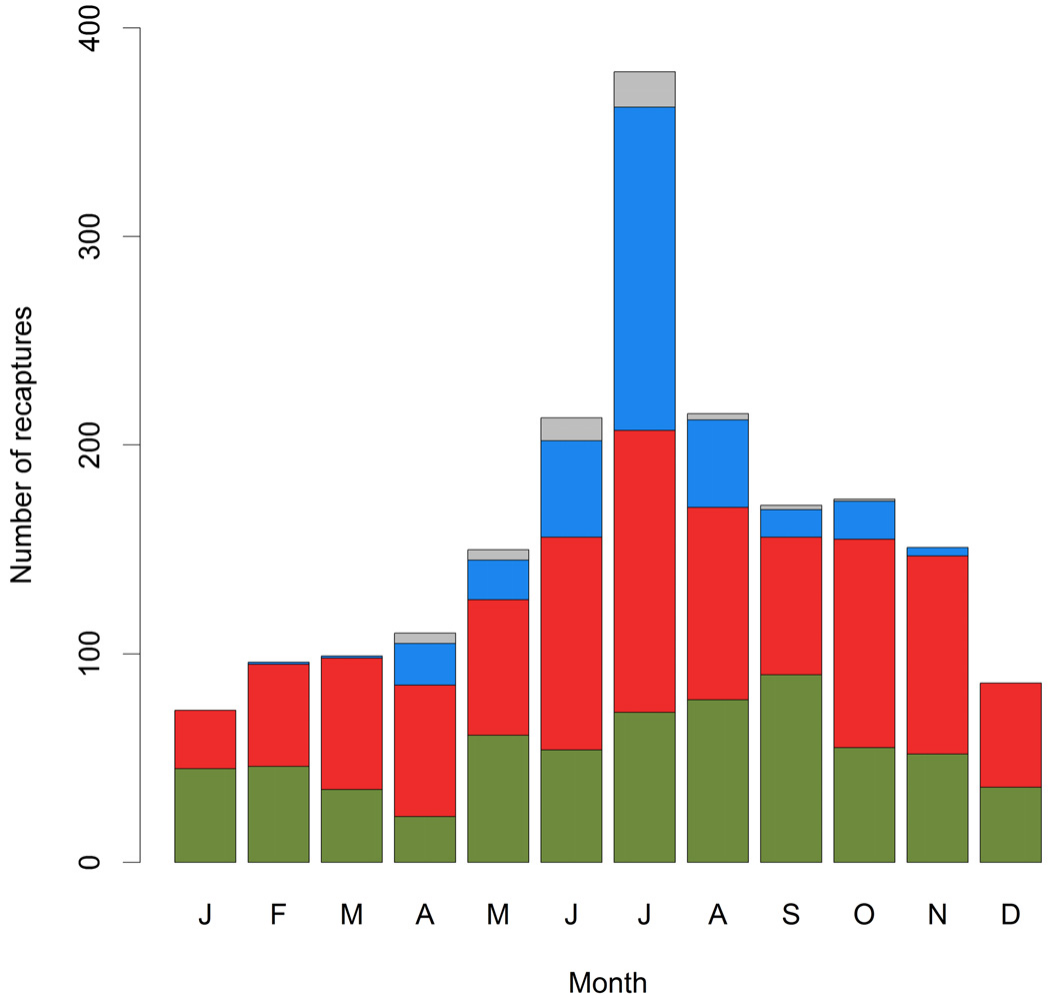
Distribution of number of dead recoveries throughout the year. Height of bars indicate number of reported fish for each month (J: January, F: February and so on). Black: proportion caught by professional fishermen. Grey: recreational fishery. Diagonally shaded area is proportion taken by recreational fishermen from the local area. Vertical shaded areas represent fish reported by recreational fishermen with postal codes in other parts of Norway. Unshaded grey areas represent fish reported by foreign recreational fishermen.

During 2005–2013 a total of 9 729 cod (mean length: 40.7 cm, range: 16–93 cm) were tagged and released. By the end of 2013 a total of 1139 (11.7%) cod were reported as harvested by recreational fishers while a total of 568 (5.8%) cod were reported harvested by commercial fishers (Table 2). Only 49 fish were reported recaptured outside the defined study area (of which at least 13 were recovered in a country outside Norway). The peak season was summer from June to August, covering 42% of total reported recaptures (Fig 3).

**Table 2:** Tag returns (low = 50 NOK, high = 500 NOK) per year from 2005–2013 from commercial fisheries and recreational rod/line, fixed and unknown gear. Despite recoveries occurred at any time, including 2005, data formatting for the mark-recovery model require individual deaths reported soon after tagging and/or during the time interval between years to be entered in the next season (for details, see materials and methods).

	2005	2006	2007	2008	2009	2010	2011	2012	2013	Total
Commercial high	0	11	15	25	17	16	8	12	15	119
Commercial low	0	47	118	110	82	23	25	9	35	449
Rec line high	0	7	9	16	18	13	14	19	15	111
Rec line low	0	35	36	34	38	16	19	31	20	229
Rec fixed high	0	8	5	34	16	10	4	7	21	105
Rec fixed low	0	60	58	87	59	19	17	10	17	327
Rec unknown high	0	14	14	11	13	4	7	2	0	65
Rec unknown low	0	82	96	61	37	13	6	7	0	302

Recreational and commercial fishers reported using a variety of fishing gear. Recoveries by commercial fishers were mainly caught by fyke net and gill net. Recreational catches were divided into rod and line, fixed gear and unknown gear, representing mean respective proportions of 22.2, 23.8 and 20.8% of all recoveries (Σ = 66.7%), and 3.5, 4.4 and 3.7% of all tagged cod over the 9 year study period (Table 2). Length measurements were reported by 67% of recreational fishers and 69% of commercial fishers. Mean length of cod reported by recreational fishers were 47.3 cm (SE 0.7) for handline and 49.3 (SE 0.7) for gillnet. For cod reported by commercial fishers the mean length was 52.5 cm (SE 0.5).

Local residents accounted for 71.4% of all reported recreational recaptures. Norwegian tourists were the second largest contributor with 25.1% of recaptures. Foreign tourists were responsible for only 3.4% of reported recreational recaptures. The peak in recreational catches was in July, during which 24% of all recreational recoveries were reported (Fig 3). However, reported tag recoveries by foreign tourists and local residents also peaked in July. Tag recoveries from commercial fishers were more spread out through the year (Fig 3).

### Model results

#### Model fit and selection

The GOF tests performed for this set of data yielded significant results (lack of fit of the reduced 2-state model) for the low reward tagging group (Table 3). However, the ĉ coefficient resulting from the global test was close to 1, indicating low overdispersion of the data. In the multi-event modeling we departed from a more complex model (model 1, table of models) that considered different sources of mortality and accounted for time and reward effects, so incorporating many potential sources of lack of fit. We began model selection by focusing on the survival parameter (S) and increased model parsimony by removing time effects (model 2 vs. model 1). Then, we kept this structure to proceed with the modelling of mortality proportions (M); however, removing time effects from fishing mortality proportions was not well supported (model 3 vs. model 2) so we eventually retained model 2 as the best structure in the set (Table 4).

**Table 3:** Global test results of the Goodness-of-fit (GOF) test performed for the reduced 2-state model with single live and newly dead states (see methods).

Data	Chi-square	*P*	df	ĉ
Group 1 (500 NOK reward)	18.587	0.136	13	1.429
Group 2 (50 NOK reward)	33.651	0.009	17	1.979
Sum over groups	52.238		30	**1.741**

**Table 4:** Model selection towards a consensus model for survival (*S*) and fishing mortality proportions (*M*). Reporting rates (*r*) were always kept as function of fishery type (gear) and reward. In the case of unknown gear types, two periods of constant but different reporting rates were also included in the model structure (see methods). Departing from the general full time-dependent structure (model 1), modelling consisted in removing time effects (time) first from survival (model 2) and secondly, from mortality proportions (model 3) keeping the best structure in the survival parameter. Resulting models were ranked according to QAIC values (see methods). Deviance and number of parameters (Np) are also given. Delta QAIC indicates the difference in QAIC between the current model and the final retained model. The best model is shown on top.

Model	S	M	r	Np	Deviance	QAIC	DeltaAIC
2	(.)	gear*time	gear*reward	37	15742.6091	9116.2798	0
1	time	gear*time	gear*reward	43	15734.4341	9123.5842	7.3044
3	(.)	gear(.)	gear*reward	9	16042.6076	9232.5937	116.3139

#### Reporting rates, annual survival and mortality proportions

In general, high-reward tags were reported with more probability than standard tags (Table 5). However, reporting rate of deaths due to recreational lines were the lowest, and model estimates indicate that only a 19% of high-reward captures in this fishery were reported (Table 5).

**Table 5:** Reporting rate per fishery type and reward (High = 500 NOK, low = 50 NOK).

Type of Recovery	Tag reward	Estimate	CI-	CI+	SE
Commercial fisher	High	0.99933204	0.9993296	0.99933448	1.245E-06
Recreational line	High	0.19107789	0.13541537	0.26266932	0.03240239
Recreational fixed gear	High	0.99998133	0.9999798	0.99998274	7.51E-07
Commercial fisher	Low	0.72761128	0.50778186	0.87368527	0.09620524
Recreational line	Low	0.08559838	0.06089735	0.11904845	0.01466115
Recreational fixed gear	Low	0.65577818	0.44706842	0.81781228	0.09870746

Model results also indicated that reporting of unknown recreational gear catches decreased towards the end of the study period (Table 6); in this case, reporting rates for each of the tagging groups were similar, but slightly higher for high reward tags.

**Table 6:** Reporting rates of recreational unknown gear. (High = 500 NOK, low = 50 NOK).

Type of Recovery	Tag reward	Recovery period	Estimate	CI-	CI+	SE
Unknown rec. gear	High	Before 2009	0.20747458	0.12808937	0.31810962	0.04846817
Unknown rec. gear	High	After 2009	0.04975389	0.0232519	0.10326861	0.01901292
Unknown rec. gear	Low	Before 2009	0.15305057	0.10864452	0.211304	0.02604357
Unknown rec. gear	Low	After 2009	0.02997352	0.01675804	0.05304851	0.0088259

General survival of cod in our study area was low (Table 7) and cause-specific mortality proportions varied over time, but estimates derived from the retained model indicate that fishing activities may represent, on average, a 56% of total annual mortality of cod in fished areas in Skagerrak; this percentage results from summing the 15% associated to the commercial harvest with the mortality proportions associated to recreational line and fixed gears (see Table 7). Considering only fishing-related deaths, recreational rod and line accounted for a 60% of the total fishing mortality.

**Table 7:** General survival and mortality proportions from commercial fisheries and recreational line and fixed gear. Natural mortality is lower than fishing mortality.

Parameter	Estimate	CI-	CI+	SE
General survival	0.32618198	0.29707416	0.35669459	0.01522419
% deaths due to commercial fisheries	0.15136585	0.13614315	0.16795972	0.00811174
% deaths due to recreational line	0.33680026	0.29222837	0.38447745	0.02359014
% deaths due to recreational fixed	0.06751096	0.05976538	0.07617904	0.00417982
% deaths due to other causes (natural)	0.44432293	0.39673935	0.49295009	0.02461873

## Discussion

Nine years of tag-recovery data covering 100 km of Skagerrak coastline suggests that 72% of harvested coastal Atlantic cod was caught by recreational fishers. Recreational fishers used a variety of gear to capture cod, including rod and line, traps and gill net. Rod and line fishing appeared to have high impact, accounting for 60% of total fishing mortality. Our findings are in line with other studies that have found that fishing mortality from recreational fishing can be higher than from the commercial sector [3, 5, 35, 36]. The southern Norway coastline is a popular fishing area for recreational purposes in summer [22], as well as a popular tourist destination during summer. Our results reflect the increased fishing activity in summer. For Norwegian tourists, 71% of recoveries were reported between June and August.

We have based our analysis on the assumptions that (1) tagged samples were representative of the coastal cod population, (2) tagged cod mixed with untagged cod in the areas, and (3) the tag recoveries were reported correctly [30]. One of the strengths with the model presented herein is that we were not dependent on the assumption of 100% return rate of high reward tags [37]. There is reason to argue that foreign tourists had a lower likelihood of reporting high reward tags due to language problems, cultural differences, transience and unfamiliarity with the tagging program. In addition, some tags were reported by local fish camps. It is known that fish camps harbour a high proportion of foreign tourists [8]. We therefore expected underestimation of catches made by foreign tourists. Reporting rate by tourists compared to local residents was not analysed.

The present study had one annual tagging period lasting from April to July. Recreational recaptures peaked in summer, while commercial recaptures were spread throughout the year. One tagging period, in our case conducted just before the peak recreational fishing season, might potentially have lead to an overestimate of recreational catches compared to commercial. Future studies should therefore include more than one tagging period in order to test this potential bias [27]. Herein we focused on coastal Atlantic cod and tagging was performed from inshore locations (e.g., inside fjords) to slightly more exposed locations within coastal archipelagos. Our tagging effort covered cod in more exposed off-shore areas to a limited extent only. The current standing hypothesis is that North Sea eggs and larvae drift from spawning areas in the North Sea with the current into Skagerrak, where they settle mostly in exposed areas of the coastline [38, 39, 40]. Cod in these areas resemble North Sea cod genetically [41] and seem to have slightly more active movement behaviours in which long distant migrations are more frequent [16]. Cod from areas further off the coast, usually caught by commercial vessels using trawl and gillnet are probably underrepresented in this study. However, according to recent genetic findings based on samples collected over several years from exposed parts of the coastline, it is expected that Atlantic cod caught in these offshore areas do not originate from local coastal cod populations [41]. Cod is the primary by-catch species in the offshore shrimp (*Pandalus borealis*) fishery in Skagerrak. Out of 568 commercial recoveries reported in this study, only 15 were reported from shrimp trawlers, despite the fact that these are particularly active. This observation lends further support to the genetic evidence for stock separation, for which natal homing has been suggested as the primary separating mechanism [40]. It is therefore important to note that this study is restricted on catches of cod in coastal areas. There are no available data on geographic effort distribution for neither recreational nor commercial fishers. Potential off-shore fisheries, both recreational and commercial, are therefore expected to be underestimated. However, as mentioned above, most cod captured off-shore resembles the North Sea cod [41] and thus the off-shore fishing is not expected to have a high impact on local coastal cod populations. Further, the length measurements reported by fishers indicate that commercial fishers catch larger cod than their recreational counterparts. There is a potential that the commercial fishers’ targeting of larger cod than the recreational fishers might lead to a bias if the size distribution of tagged cod is targeted more heavily by one group. At present, there are no available data to compare the representative length differences in commercial or recreational fisheries. Further, the variety of fixed gear in use (different trap/pot designs, trammel nets and various mesh sizes in gillnets) makes access to data on gear selectivity challenging. However, there is a risk for a bias in the actual reported lengths by fishers. This bias may differ between commercial and recreational fishers. The self-reported cod lengths provided by fishers are wrought with some uncertainty, and the data should thus be treated with caution.

In a survey conducted in the German part of the Baltic Sea, Strehlow et al. [42] found that the magnitude of recreational fishing in terms of catches varied from 34 to 70% of the German commercial landings among the study years. Sparrevohn and Storr-Paulsen [43] estimated that recreational catches amounted to 4.8% of the total cod catches in Denmark. The results presented herein strengthen the notion that recreational fishing is a significant and important part of fishing mortality in northern Europe. The fishing opportunities found in Norway attracts a large number of fishing tourists from overseas, such as Germany and Denmark [8].

Kleiven et al. [5] found that recreational fishers in coastal Skagerrak account for 65% of the total catches of European lobster (*Homarus gammarus*). Both cod and lobster can be seen as important target species for recreational and commercial fishers in coastal Skagerrak. Norway does not have a monitoring system in place to estimate recreational catches. The domination of catches by recreational fishers, catches that are not monitored, indicates a flaw in the Norwegian management system. Our findings confirm the need to include recreational fisheries as an important mortality factor along the Norwegian coast. By mainly targeting commercial fisheries in data collection and management efforts, a significant part of the fishing mortality is likely to be sustained by recreational fisheries and overlooked by management.

## Supporting Information

S1 Encounter History Dataset Each row in the dataset contains information regarding a single individual. (TXT)

S1 Text File specification of the multi-event modelling approach in program E-SURGE. Description of the model pattern and matrices used in the analysis of recovery data.
(DOC)
