# Correction: Harvest Pressure on Coastal Atlantic Cod (*Gadus morhua*) from Recreational Fishing Relative to Commercial Fishing Assessed from Tag-Recovery Data

**DOI:** 10.1371/journal.pone.0159220

**Published:** 2016-07-08

**Authors:** Alf Ring Kleiven, Albert Fernandez-Chacon, Jan-Harald Nordahl, Even Moland, Sigurd Heiberg Espeland, Halvor Knutsen, Esben Moland Olsen

The figure legend for Fig 3 is incorrect. Please see the corrected Fig 3 here.

**Fig 3:**
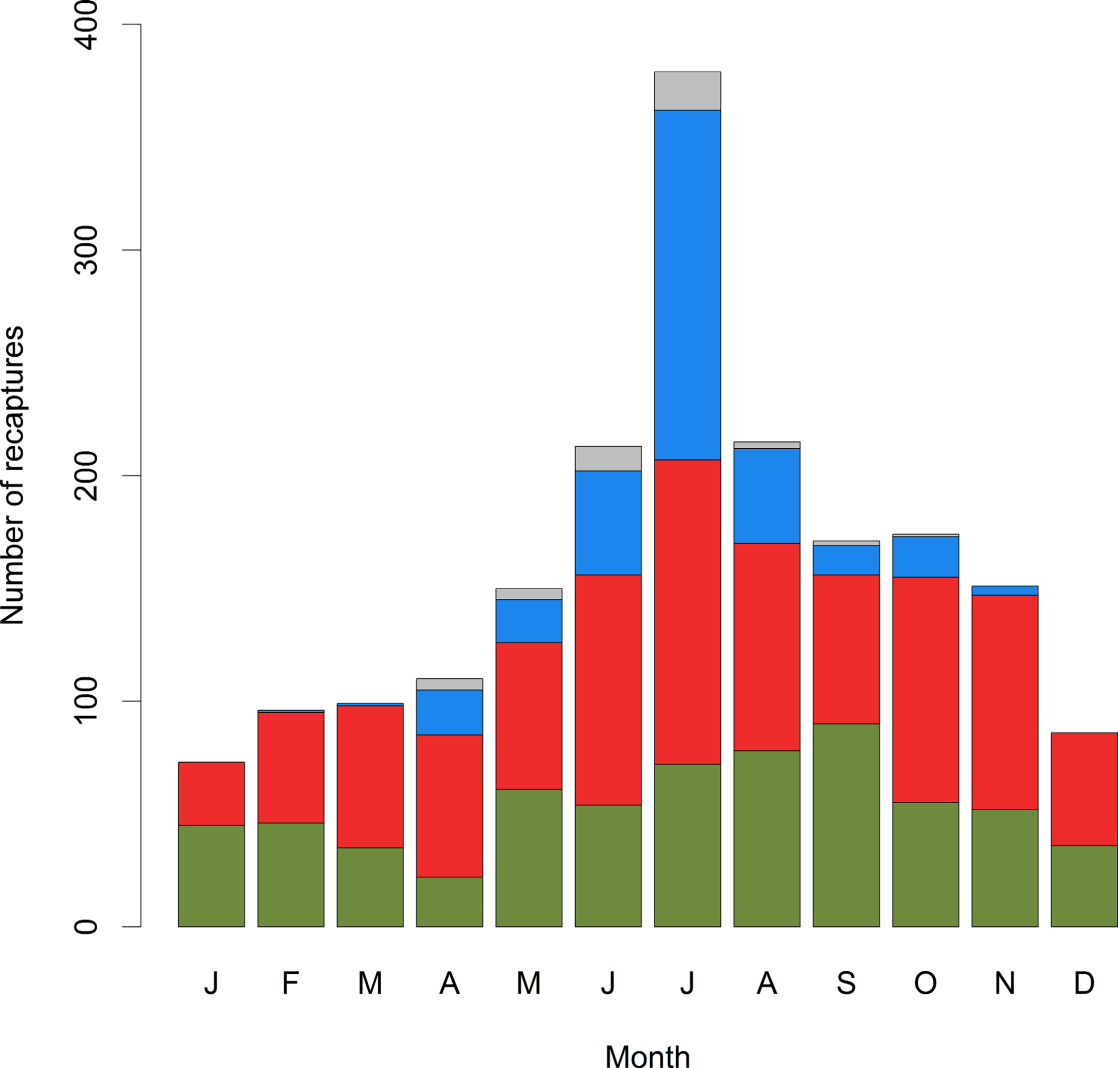
Distribution of number of dead recoveries throughout the year. Height of bars indicate number of reported fish for each month (J: January, F: February and so on). Green: proportion caught by professional fishers. Red: Recreational fishers from the local area. Blue: Recreational fishers with postal codes in other parts of Norway. Grey: Fish reported by foreign recreational fishers.
