# Supplementary material for: Harvest Pressure on Coastal Atlantic Cod (Gadus morhua) from Recreational Fishing Relative to Commercial Fishing Assessed from Tag-Recovery Data
Source: PLoS One. 2016 Mar 9;11(3):e0149595. doi: 10.1371/journal.pone.0149595 (PMC4784990; doi:10.1371/journal.pone.0149595)
Supplement: S1 Text — Description of the model pattern and matrices used in the analysis of recovery data. (DOC) [file pone.0149595.s002.doc]

**Supporting information: Text file S1**

**Decomposing mortality processes and model implementation in program E-SURGE**

Multi-event models were built in several stages using software program E-SURGE (Choquet and Nogue 2010). Using the GEPAT (GEneration of PATterns) tool implemented in program E-SURGE, the transition matrix **Φ** (see text) was decomposed into two matrices, **T1** and **T2**, each describing a specific process, or step, of the transition across states so that **Φ = T1*T2**. The first matrix, **T1**, describes the survival process:

**T1**=

| From/to | L | D | † |
| --- | --- | --- | --- |
| L | *S* | *(1-S)* | 0 |
| DC | 0 | 0 | 1 |
| DL | 0 | 0 | 1 |
| DS | 0 | 0 | 1 |
| DO | 0 | 0 | 1 |
| † | 0 | 0 | 1 |

where the probability of moving from state “L” at time *t* to state “L” at time *t+1* (i.e. to remain alive) is defined by the survival parameter *S*. Dead individuals cannot come back to life, so individuals in states “DC”, “DL”, “DS” and “DO” at time *t* do not have any survival probability and in the next time step they can only belong to the “long-time dead” state (†), with a probability of 1. Total mortality (*1-S*) is estimated by transitions from state “L” to an intermediate “newly dead” state denoted “D”. Note that “D” is not stratified according to the different causes of mortality. This is done in the last matrix, **T2**, which describes the process associated with the cause of death:

**T2**=

| From/to | L | DC | DL | DS | DO | † |
| --- | --- | --- | --- | --- | --- | --- |
| L | 1 | 0 | 0 | 0 | 0 | 0 |
| D | 0 | *m_1_* | *m_2_* | *m_3_* | *(1-m_1_-m_2_-m_3_)* | 0 |
| † | 0 | 0 | 0 | 0 | 0 | 1 |

Cause-specific mortality proportions are estimated at **T2**. These transitions are conditional on the individual having died (i.e. 1-*s*) in the previous step and are directed to the different causes of mortality (“DC”, “DL”, “DS” or “DO”). By definition, this step does not apply to living individuals, so individuals that have survived in the previous step remain alive with probability 1. Transitions to “DO” states are not represented by a parameter but rather calculated as the complement (1-*m_1_-m_2_-m_3_*). In fact, *m* corresponds to fishing mortality, *sensu stricto*, whereas its complement estimates the proportion of deaths due to other sources of mortality, excluding fishing.

Matrices **T1** and **T2** follow the model proposed by Schaub and Pradel (2004). When implementing the model in E-SURGE, one should specify a matrix with the initial conditions (see below), a 2-step transition matrix for survival and mortality processes (**T1** and **T2**) and a matrix with the detection probability of each event (**E**, in the main text). The matrix pattern and the syntax for the parametrization of the corresponding matrices to implement the model in software E-SURGE are as follows:

Syntax for step 1 : i

Pattern matrix :

* - - - -

Number of steps for transition : 2

Syntax for step 1 : i+f.t

Pattern matrix :

s * -

- - *

- - *

- - *

- - *

- - *

Syntax for step 2 : to.t

Pattern matrix :

* - - - - -

- m m m * -

- - - - - *

Number of steps for encounter: 1

Syntax for step 1 : firste+nexte.to(2)+nexte.to(3,4,5).g+nexte.to(6).t(2 3 4 5).g+nexte.to(6).t(6 7 8 9).g

Number of shortcuts : 0

Pattern matrix :

* p - - - -

* - r - - -

* - - r - r

* - - - r r

* - - - - -

* - - - - -

When building the pattern matrices in E-SURGE, each row corresponds to a multinomial. Consequently, the total of cell probabilities is 1. Because of this constraint, one and only one cell probability in each row will be calculated as the complement to 1 of the others (i.e. 1-∑ others). This particular cell is denoted with a ‘*’ symbol. Inactive cells, (i.e. cells whose associated probability is structurally 0) are denoted with a ‘-’ symbol. An active cell receives an arbitrary letter. Note that the same letter/symbol in two cells does not mean that the two values should be equal.
